# Web Alert: Vaccines against infectious agents: An annotated selection of World Wide Web sites relevant to the topics in microbial biotechnology

**DOI:** 10.1111/1751-7915.13760

**Published:** 2021-02-17

**Authors:** Lawrence P. Wackett

**Affiliations:** ^1^ Department of Biochemistry, Molecular Biology & Biophysics BioTechnology Institute University of Minnesota St Paul MN 55108 USA

## Biotechnology accelerating vaccines

https://www.nature.com/articles/d41586‐020‐02154‐2

This *Nature* news article deals with new biotechnology facilitating vaccine development with an emphasis on RNA‐based vaccines and vaccines directed against SARS‐CoV‐2.

## Historical development of hepatitis B vaccine

https://www.sciencedirect.com/science/article/pii/S1369848617300857

This article covers the history of the development of the hepatitis B vaccine between 1968 and 2000, including the development of a recombinant vaccine in 1986.

## Explanation of lipid‐nucleic acid pharmaceuticals

https://qz.com/1948132/the‐first‐covid‐19‐vaccines‐have‐changed‐biotech‐forever/

This news‐type article highlights the delivery of nucleic acid therapeutics encapsulated in lipid particles that allow more targeted delivery than small molecule drugs. The treatment includes current coronavirus vaccine development.

## Covaxin; different vaccine against coronavirus

https://www.nytimes.com/interactive/2021/health/bharat‐biotech‐covid‐19‐vaccine.html

While the most rapidly developed SARS‐CoV‐2 vaccines use RNA technology, this article describes a vaccine developed by using beta‐propiolactone to kill the virus particles that constitute the antigen.

## Three biotech advances for new advances

https://builtin.com/healthcare‐technology/biotechnology‐effective‐coronavirus‐vaccine

This article, written at the start of the 2019 coronavirus pandemic, describes the different types of vaccines than can now be developed: genetically engineered viruses, DNA vaccines and mRNA vaccines.

## First Ebola vaccine approved

https://www.nature.com/articles/s41587‐019‐0385‐7

The first Zaire Ebola virus vaccine was approved in November, 2019. The vaccine uses a vesicular stomatitis virus that expresses an Ebola surface glycoprotein.

## New vaccine technologies

https://www.frontiersin.org/articles/10.3389/fimmu.2018.01963/full

This is a journal review article that discusses viral vector, DNA and m‐RNA based vaccines.

## Bacterial vaccine production

https://www.cell.com/trends/biotechnology/fulltext/S0167‐7799(19)30050‐2

This review article deals specifically with the development of vaccines directed against pathogenic bacteria. It proposes the application genomic methodologies to accelerate the process of strain selection for vaccine development.

## Inactivated‐virus vaccines

https://www.bharatbiotech.com/blog/vaccines/the‐emergence‐of‐inactivated‐vaccines/

This blog on a commercial website covers types of vaccines: inactivated pathogens, live‐attenuated pathogens, sub‐unit and nucleic acid‐based, and toxoid vaccines.

## Reverse vaccinology

https://www.frontiersin.org/articles/10.3389/fimmu.2018.02315/full

This comprehensive review article deals largely with bacterial vaccines and also discussed the use of genomics in strain selection and the use of monoclonal antibodies.

## Bacterial capsular polysaccharide antigens

https://www.cytivalifesciences.com/en/us/solutions/bioprocessing/knowledge‐center/bacterial‐vaccine‐manufacturing

This webpage describes the characterization and purification of bacterial capsular polysaccharides for use in vaccine production.

## Vaccine simplicity

https://weekly.biotechprimer.com/vaccines‐powerful‐simplicity/

This news article describes the different types of SARS‐CoV‐2 virus vaccines that have been worked on and the companies that are doing that research.

## Principles of inactivation

https://www.cdc.gov/vaccines/pubs/pinkbook/prinvac.html

This government website deals with vaccines in the context of the human immune response.

## History of vaccines

https://www.historyofvaccines.org/index.php/content/articles/different‐types‐vaccines

This webpage covers a history of vaccine development and the principle organisms and viruses the vaccines are directed against.

## Vaccine manufacturing: Lonza

https://bioscience.lonza.com/lonza_bs/US/en/vaccine‐manufacturing

This commercial website deals with the process of vaccine manufacturing.

## Standards for vaccines

https://www.usp.org/sites/default/files/usp/document/our‐impact/covid‐19/standards‐for‐quality‐vaccines.pdf

Vaccine manufacture requires standard reagents and procedures. This website deals with these methods and materials that constitute quality control for vaccine development.

## Recombinant vaccine compendium

https://www.nature.com/subjects/recombinant‐vaccine

This *Nature* research page offers a compendium of references and links to further information on recombinant vaccines.

## Different types of vaccines

https://www.news‐medical.net/health/What‐are‐the‐Different‐Types‐of‐Vaccines.aspx

This article describes the different types of vaccines and the microorganisms and viruses that each are used with.

## Recombinant influenza vaccines

https://www.frontiersin.org/articles/10.3389/fimmu.2019.02997/full

This article discusses the influenza hemagglutinin vaccine. This article describes efforts to overcome the variability of the hemagglutinin head region to stay one step ahead of the virus.

## Influenza vaccines

https://www.ncbi.nlm.nih.gov/books/NBK537197/

This article deals with the mechanism of action and administration of influenza vaccines.

## Work towards development of HIV vaccine

https://www.hiv.gov/hiv‐basics/hiv‐prevention/potential‐future‐options/hiv‐vaccines

This website describes efforts to develop a vaccine against HIV.

## Diphtheria‐Tetanus‐Pertussis (DPT) vaccine

https://www.ncbi.nlm.nih.gov/books/NBK545173/

The DPT vaccine is comprised of inactivated diphtheria and tetanus toxins and pertussis antigens.

## Tetanus prophylaxis

https://www.ncbi.nlm.nih.gov/books/NBK559008/

Possible exposure to the toxin produced by *Clostridium tetani* requires a tetanus vaccine, as described in detail here.

## Glycoconjugate vaccine design

https://pubmed.ncbi.nlm.nih.gov/33388125/

This paper deals with vaccine development using the *Streptococcus* type III capsular polysaccharide.

